# Biochemical Response and Gene Expression to Water Deficit of Croatian Grapevine Cultivars (*Vitis vinifera* L.) and a Specimen of *Vitis sylvestris*

**DOI:** 10.3390/plants12193420

**Published:** 2023-09-28

**Authors:** Katarina Lukšić, Ana Mucalo, Ana Smolko, Lidija Brkljačić, Luka Marinov, Katarina Hančević, Maja Ozretić Zoković, Marijan Bubola, Edi Maletić, Jasminka Karoglan Kontić, Marko Karoglan, Branka Salopek-Sondi, Goran Zdunić

**Affiliations:** 1 Institute for Adriatic Crops and Karst Reclamation, Put Duilova 11, 21000 Split, Croatia; katarina.luksic@krs.hr (K.L.); ana.mucalo@krs.hr (A.M.); luka.marinov@krs.hr (L.M.); katarina.hancevic@krs.hr (K.H.); maja.ozretic@krs.hr (M.O.Z.); 2 Ruđer Bošković Institute, Bijenička 54, 10000 Zagreb, Croatia; ana.smolko@irb.hr (A.S.); lidija.brkljacic@irb.hr (L.B.); branka.salopek.sondi@irb.hr (B.S.-S.); 3 Institute of Agriculture and Tourism, Karla Huguesa 8, 52440 Poreč, Croatia; marijan@iptpo.hr; 4 Department of Viticulture and Enology, Faculty of Agriculture, University of Zagreb, Svetošimunska Cesta 25, 10000 Zagreb, Croatia; emaletic@agr.hr (E.M.); jkkontic@agr.hr (J.K.K.); mkaroglan@agr.hr (M.K.); 5 Centre of Excellence for Biodiversity and Molecular Plant Breeding, Svetošimunska Cesta 25, 10000 Zagreb, Croatia

**Keywords:** water deficit, RT-qPCR, aquaporins, ABA, SA

## Abstract

The biochemical response and gene expression in different grapevine cultivars to water deficit are still not well understood. In this study, we investigated the performance of four traditional Croatian *Vitis vinifera* L. cultivars (‘Plavac mali crni’, ‘Istrian Malvasia’, ‘Graševina’, and ‘Tribidrag’), and one wild (*Vitis vinifera* subsp. *sylvestris*) genotype exposed to water deficit (WD) for nine days under semi-controlled conditions in the greenhouse. Sampling for biochemical and gene expression analyses was performed at days six and nine from the beginning of WD treatment. The WD affected the accumulation of metabolites with a significant increase in abscisic acid (ABA), salicylic acid (SA), and proline in the leaves of the stressed genotypes when the WD continued for nine days. Lipid peroxidation (MDA) was not significantly different from that of the control plants after six days of WD, whereas it was significantly lower (297.40 nmol/g dw) in the stressed plants after nine days. The cultivar ‘Istrian Malvasia’ responded rapidly to the WD and showed the highest and earliest increase in ABA levels (1.16 ng mg^−1^ dw, i.e., 3.4-fold increase compared to control). ‘Graševina’ differed significantly from the other genotypes in SA content at both time points analyzed (six and nine days, 47.26 and 49.63 ng mg^−1^ dw, respectively). Proline level increased significantly under WD (up to 5-fold at day nine), and proline variation was not genotype driven. The expression of aquaporin genes (*TIP2*;*1* and *PIP2*;*1*) was down-regulated in all genotypes, coinciding with the accumulation of ABA. The gene *NCED1* (9-cis-epoxycarotenoid dioxygenase) related to ABA was up-regulated in all genotypes under stress conditions and served as a reliable marker of drought stress. This work suggests that the stress response in metabolite synthesis and accumulation is complex, treatment- and genotype-dependent.

## 1. Introduction

Recent climate changes, characterized by increased temperatures and exacerbated droughts, are having a significant impact on various agricultural sectors, particularly in the semi-arid and arid Mediterranean regions [1]. Unfortunately, future projections indicate even more severe drought conditions in the Mediterranean region [2,3]. Although grapevine (*Vitis vinifera* L.) is generally well adapted to dry conditions [4,5], it can suffer significant tissue damage and reduce fruit quality and yield under prolonged drought conditions.

The morphological signs of drought stress often become apparent only after significant damage has already occurred. Therefore, it is important to identify reliable markers of drought stress at the physiological, biochemical and genetic levels [6]. Phytohormones play an important role in plant response to drought stress. Among them, abscisic acid (ABA) is considered the most important hormone associated with drought stress. ABA has a rapid effect on regulating stomatal closure, which is crucial for water conservation [7,8,9,10]. Under mild drought conditions, ABA has a protective effect, whereas under severe stress it shows negative effects through prolonged or complete stomatal closure and leaf senescence [7,9,11]. Genes involved in the biosynthesis of ABA, such as *NCED1* and *NCED2* (9-cis-epoxycarotenoid dioxygenase), have been identified as reliable markers of drought stress [8,12], although there are limited data on the expression of *NCED1* and *NCED2* in different grapevine cultivars during the water deficit.

Changes in the hydraulic pathway, including regulation of aquaporins (AQPs), are also involved in the response to water deficit stress. AQPs are cell membrane proteins that facilitate the transport of water and small molecules across plasma membranes. The expression patterns of individual AQP genes and their involvement in isohydric/anisohydric strategies under drought conditions have not been fully elucidated [13]. Tonoplast intrinsic proteins (TIPs) and plasma membrane intrinsic proteins (PIPs) of the AQP family have been shown to respond to drought in grapevines [14]. Genes such as *TIP2*;*1* and *PIP2*;*1* are particularly important for regulation of water transport under drought conditions [9,15]. In addition to hormonal and hydraulic responses, plants also employ osmotic adjustment mechanisms to cope with drought stress. Osmolytes, such as proline, play a crucial role in reducing cell water potential and allow the uptake of water from the soil. Reactive oxygen species (ROS) accumulation during drought stress can lead to oxidative stress, causing damage to DNA, lipids, proteins, and cell membranes [8].

One approach to adapt to climate change is the utilization of traditional grapevine cultivars that are adapted to specific environmental conditions. However, there is a lack of studies investigating the molecular variability related to water regulation in traditional cultivars. Adaptation to drought of traditional cultivars, or cultivars specific to a certain region, were mostly examined from phenological or physiological perspectives [16,17,18,19,20]. Recent studies, broadened with molecular responses, provide a clearer picture of grapevine behavior under drought and enable detecting water stress signals at early stages [11,21,22,23,24]. Understanding the responses of grapevines to water deficit stress is crucial for developing effective strategies to enhance its resilience to drought.

In this study, we aimed to investigate the effects of water deficit at two time-points (six and nine days) on four traditional Croatian grapevine cultivars (‘Graševina’, ‘Plavac mali crni’, ‘Istrian Malvasia’, and ‘Tribidrag’) and one wild *Vitis vinifera* subsp. *sylvestris* (hereafter referred to as *V. sylvestris*) genotype. These cultivars were grown on their own roots in a greenhouse in 5-L pots. ‘Graševina’ is the predominant cultivar in the continental part of Croatia, whereas ‘Istrian Malvasia’ and ‘Plavac mali crni’ are commonly grown in the northern (Istria) and southern (Dalmatia) coastal regions, respectively. ‘Tribidrag’ is regaining importance in Croatia due to its recent genetic discovery of synonymy with the American cultivar ‘Zinfandel’ and the Italian cultivar ‘Primitivo’. The *V. sylvestris* differs to some extent from cultivars in terms of its genomic constitution, and information about its drought tolerance is limited. We conducted metabolite profiling focusing on ABA, SA, MDA and proline, and analyzed the expression of selected genes related to AQP and ABA biosynthesis. This is the first research on the response of different grape varieties to drought in Croatia.

## 2. Results

### 2.1. Biochemical Stress Markers

Stress-related metabolites (ABA, SA, MDA and proline) were analyzed in five grapevine genotypes at two time points (six and nine days) of water deficit treatment.

A two-way ANOVA analysis revealed a significant genotype and treatment effect at both time points on metabolite accumulation during water deficit (Table 1). A significant genotype-by-treatment interaction (G × T) was found only for ABA and proline six days upon water deficit (*p* < 0.001).

**Figure 1:**
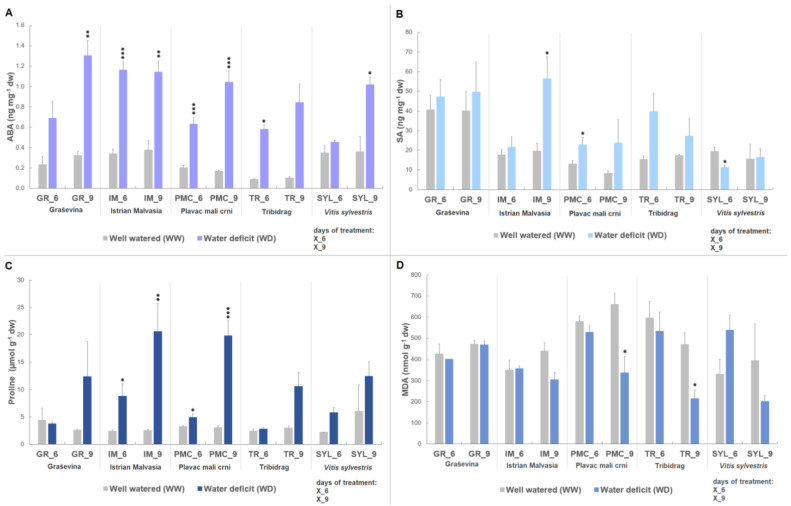
Stress-related metabolites in leaves of four *Vitis vinifera* L. cultivars (‘Graševina’—GR; ‘Istrian Malvasia’—IM; ‘Plavac mali crni’—PMC and ‘Tribidrag’—TR); and one wild *V. sylvestris*, SYL subjected to water deficit treatment (WD) at two time points (day six and nine) compared to the corresponding well-watered controls (WW): (**A**) abscisic acid content (ABA, ng mg^−1^ dw); (**B**) salicylic acid content (SA, ng mg^−1^ dw); (**C**) proline content (µmol g^−1^ dw); and (**D**) lipid peroxidation (MDA, malondialdehyde, nmol/g dw). Asterisks in the graphs indicate significant accumulation of metabolites compared with the corresponding control for each genotype (Student’s *t*-test, * represents *p* < 0.05, ** *p* < 0.01, *** *p* < 0.001). Bars show means + SE of three biological replicates.

After six days of stress, IM had significantly higher ABA content (1.6- to 1.9-fold) compared with the other genotypes and, together with PMC, showed early accumulation under stress (Figure 1A). At the same time point, GR had significantly higher SA content (1.5- to 3-fold) (Table 1), followed by TR. TR also had significantly higher MDA content (up to 1.6-fold), followed by PMC and SYL. Proline showed a significant increase of 1.8-fold at WD, but no significant changes were observed between genotypes.

At nine days, increase in ABA (3.9-fold) and proline (5-fold) under WD showed to be only treatment-dependent. SA was still significantly higher in GR (1.4- to 3-fold) compared with the other genotypes. MDA accumulation was mainly genotype driven in this experiment. MDA content between treatments (WW vs. WD) significantly differed only upon nine days of water deficit (~0.6-fold decrease in WD). The highest MDA content between genotypes upon nine days was observed in PMC (Table 1), followed by GR and IM. 

At both time points, water deficit led to a significant increase in ABA, SA and proline, while a significant decrease was observed for MDA only, at the second time point.

When control and water deficit treatments were compared between genotypes, there was a significant increase in ABA content at IM, PMC, and TR at six days (Figure 1A). This increase was permanent in the second time point at nine days, except for TR. Other genotypes, GR and SYL, showed a significant increase in ABA (4-fold and 2.8-fold, respectively) only at nine days. PMC and SYL showed significant differences in SA content, compared to their WW control (Figure 1B) at the sixth day. As with PMC, a significant increase (1.7-fold) in SA was observed following six days of water deficit, while SYL showed a decrease (0.6-fold) in SA. After nine days, only IM-stressed vines showed a significant increase (2.9-fold) in SA.

IM and PMC showed significantly higher proline content in vines with water deficit at six (IM: 3.6-fold increase/PMC: 1.5-fold increase) and nine days (IM: 8-fold increase/PMC: 6.4-fold increase) (Figure 1C). A significant decrease (~0.5-fold) in MDA was observed only in PMC and TR at day nine (Figure 1D).

Results for the biochemical markers normalized to the corresponding controls are shown in Figure S1.

### 2.2. Expression of Selected Genes under Water Deficit

**Figure 2:**
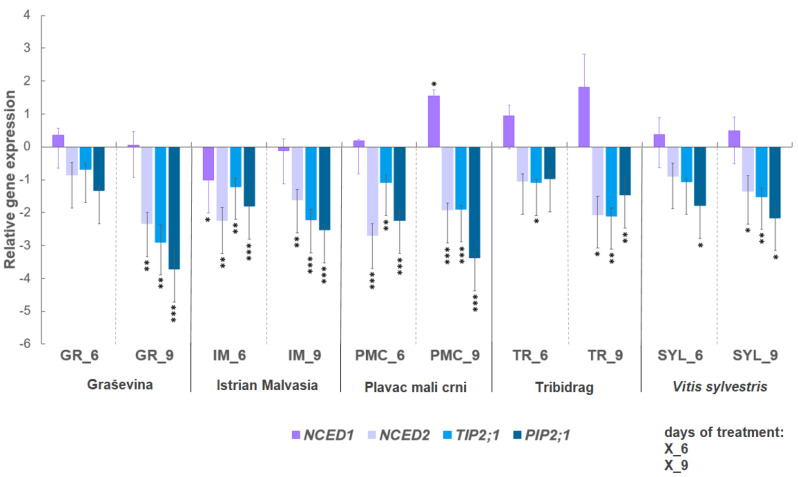
Relative gene expression of: 9-cis-epoxycarotenoid dioxygenase 1 (*NCED1*, VIT_19s0093g00550); 9-cis-epoxycarotenoid dioxygenase 2 (*NCED2*, VIT_10s0003g03750); aquaporin *TIP2*;*1* (VIT_09s0002g04020) and aquaporin *PIP2*;*1* (VIT_13s0019g04280) of four *Vitis vinifera* L. cultivars (‘Graševina’—GR; ‘Istrian Malvasia’—IM; ‘Plavac mali crni’—PMC; and ‘Tribidrag’—TR) and one wild *V. sylvestris*, SYL) subjected to water deficit at two time points (days six and nine) compared with the corresponding controls. Gene expression was calculated according to as relative gene expression compared to the control conditions. Untreated control equals one, via the definition of 2^−ΔΔCt^ method used. Asterisks indicate significant differences between water deficit treatment compared with the corresponding control of each genotype (Student’s *t*-test, * indicates *p* < 0.05, ** *p* < 0.01, *** *p* < 0.001). Bars show means ± SE of three biological replicates.

The expression level of selected genes related to the biosynthesis of ABA (*NCED1* and *NCED2*) and the AQPs (*TIP2*;*1* and *PIP2*;*1*) is shown in Figure 2, after six and nine days of water deficit compared with the corresponding control. Water deficit resulted in the down-regulation of the expression of selected genes (*NCED2*, *TIP2*;*1*, and *PIP2*;*1*) in all genotypes at both time points. Two genotypes, PMC and IM, exhibited significant down-regulation of *NCED2* at six days; earlier than the other genotypes. In addition, PMC and IM exhibited stronger signals for the down-regulation of AQP genes. *NCED1* transcript level showed a tendency to overexpress under drought conditions compared to controls, but a significant increase in expression was observed only in PMC after nine days of water deficit.

### 2.3. Principal Component Analysis (PCA)

**Figure 3:**
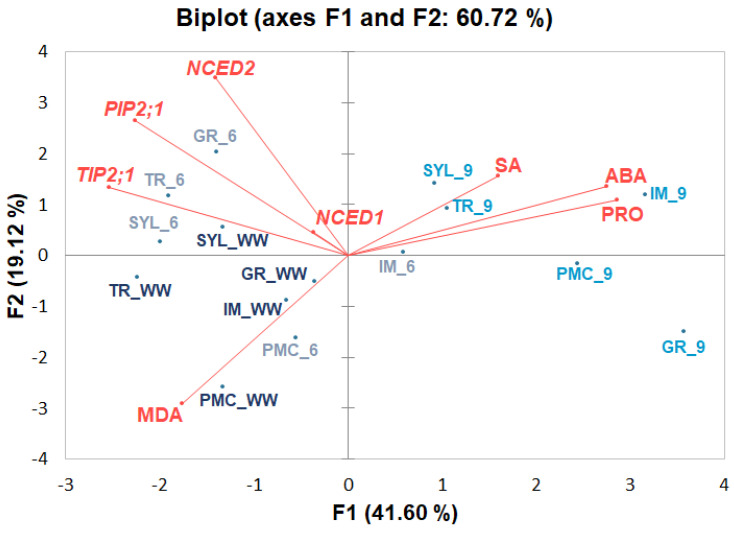
Principal component analysis (PCA) showing stress-related metabolites accompanied by selected gene expressions in four *Vitis vinifera* L. genotypes (‘Graševina’—GR; ‘Istrian Malvasia’—IM; ‘Plavac mali crni’—PMC; ‘Tribidrag’—TR) and one wild *V. sylvestris*, (SYL)) under water deficit at two time points (days six and nine) compared to the corresponding well-watered control (WW). PCA was performed on the correlation matrix of average values of stress-responsive metabolites (ABA, SA, proline (PRO) and MDA), and selected gene expression levels [9-cis-epoxycarotenoid dioxygenase 1 (*NCED1*, VIT_19s0093g00550), 9-cis-epoxycarotenoid dioxygenase 2 (*NCED2*, VIT_10s0003g03750), aquaporin *TIP2*;*1* (VIT_09s0002g04020) and aquaporin *PIP2*;*1* (VIT_13s0019g04280)].

Two-dimensional PCA analysis was performed to visualize stress-responsive metabolites along with relative expression of selected genes (Figure 3) in grapevine genotypes.

PCA used to consider the correlations between the stress-responsive metabolites, the selected gene expressions and the studied genotypes evaluated after six and nine days of drought treatment showed that PC1 accounted for 41.60% and PC2 for 19.12% of the variability and together explained 60.72% of the total variability. ABA, SA, and proline were positioned close to prolonged drought conditions (day nine) compared to controls and early drought conditions (day six of water deficit initiation). MDA was close to control and early drought response scores, indicating that no significant lipid peroxidation occurred under drought. The positioning of aquaporin genes (*TIP2*;*1* and *PIP2*;*1*) is more likely related to WD at day 6. The same could be concluded for the ABA biosynthetic genes *NCED2* and *NCED1*, whereas positioning of *NCED1* suggested its increased expression under stress, especially after six days of drought, and thus its strong contribution to the drought response compared with *NCED2*. 

The position of the genotypes under water deficit indicated that PMC and IM responded earlier to drought via accumulating stress-related metabolites, especially ABA and proline, compared with other genotypes. Prolonged water deficit resulted in high accumulation of the stress hormones SA and ABA and the osmoprotective proline in all genotypes, especially in IM, PMC, and GR. To simplify the PCA plot and for relatively similar values, the controls for both time points were pooled together as one mean value in Figure 3.

## 3. Discussion

The stress response of four grapevine cultivars (‘Graševina’, ‘Plavac mali crni’, ‘Istrian Malvasia’, ‘Tribidrag’) and one wild grapevine genotype (*Vitis vinifera* subsp. *sylvestris*) was studied at two time points, under six- and nine-days of water deficit in a greenhouse. As in previous studies [9,21,25,26], own-rooted and fruitless plants were used to obtain a genotype-specific response to water deficit. 

The variation in the accumulation of stress metabolites during the nine-day water deficit was mainly driven by treatment, indicating that all genotypes responded to the water deficit. The genotype-specific response observed in this study is well-known in grapevines, even in clones of the same cultivar [27]. 

### 3.1. Stress-Response Metabolites

The increase in ABA is one of the first signals of water deficit [28], considered a key regulator of stomatal regulation [8,29] and affecting several physiological processes at the molecular level, such as gene expression, cellular osmotic adjustment, and aquaporin activity [8]. ABA was confirmed here as a good marker of water deficit, as previously reported [12]; its signal was the strongest among the metabolites analyzed.

Five analyzed genotypes differed in the speed and persistence of ABA accumulation during water deficit. IM and PMC had an early and persistent response to water deficit, while TR showed only an early response of increased ABA accumulation. Other genotypes, GR and SYL, had a delayed response, with significantly higher levels of ABA only at the second time point (nine days). The significant increase in ABA at the first time point and the nonsignificant changes between genotypes after prolonged stress confirm ABA as one of the first signaling molecules in water deficit. After reaching the peak, de novo synthesis of ABA no longer occurs [11,30]. Degu et al. [10] observed accumulation of ABA and up-regulation of related genes between four and eight days of water deficit in grafted grapevines.

The rapid accumulation of ABA, observed for IM and PMC, is similar to that reported for the isohydric cultivar of *Triticum aestivum* [13]. Degu et al. [10] observed rapid metabolic changes within the first four days of deficit irrigation; a marked decrease in stomatal conductance was accompanied by an increase in leaf ABA content. After prolonged stress (nine days), IM showed a slight tendency to decrease ABA content in leaves. A decrease in ABA content after prolonged water deficit and its fluctuations during stress were previously observed in grapevines [11]. 

The correlations between an increase in endogenous ABA content and drought tolerance of plants are somewhat controversial in the literature and obviously depend on plant species, cultivar, and developmental stage. Positive correlations between ABA content and drought tolerance were found in sunflower and switchgrass, suggesting that constitutively high ABA content in tolerant cultivars confers an improved ability to cope with water deficit [31]. On the other hand, a comparative study of some native species from arid regions showed that the highest ABA content was found in the drought-sensitive *Poa ligularis* and the lowest in the highly tolerant xerophytic species *Papostypa speciosa* [31]. Degu et al. [10] highlighted the importance of swift changes and prompt reaction of grapevines in acclimation to water deficit. 

SA is a phytohormone that plays an important role in drought tolerance via promoting the accumulation of osmolytes such as proline or maintaining antioxidant activity [32,33]. SA is a stress-signaling molecule that activates the expression of stress-sensitive genes [34]. In the present study, SA was the only metabolite that differed significantly between genotypes and treatments throughout the experiment. The GR genotype had the highest SA level among the genotypes at both measurement time points. It has been reported that SA-accumulating mutants of *Arabidopsis thaliana* (*adr1*, *myb96-1d*, *siz1*, *acd6*, and *cpr5*) exhibit stomatal closure and improved drought tolerance [35,36]. 

Since SA is a phenolic acid with demonstrated antioxidant activity, its increased concentration in the plant could enhance a ROS-scavenging capacity [37]. However, previous studies with GR (internationally known as ‘Welschriesling’) have shown that this genotype is particularly susceptible to drought [38]. The difference between PMC and SYL in the increase and decrease in SA after six days might be related to the faster activation of other stress metabolites such as ABA, proline, or gene expression in PMC.

Proline is involved in membrane stability by supporting growth and productivity under water deficit. It is one of the most reactive amino acids produced during water deficit and, together with sugars, plays a role in osmotic adjustment [8]. The highest and earliest osmotic adjustment via proline accumulation was shown by IM and PMC compared to their controls. The significant effect of treatment on proline accumulation in this experiment indicates the efforts of all genotypes to maintain or restore osmotic balance. Although proline appears to play only a marginal role in osmotic adjustment [28], it has been reported that it may facilitate long-term adjustment to drought through keeping stomata active and open to some degree during drought [8].

Malondialdehyde (MDA), a product of lipid peroxidation, is an indicator of oxidative stress, which is expected to be high during drought [21]. In our study, the variation in MDA was mainly genotype driven. However, we observed that the MDA content at WD decreased significantly after nine days, indicating a higher overall antioxidant activity of grapevine genotypes and a higher efficiency in scavenging ROS molecules [8,22,28]. 

The antioxidant activity could be enhanced by the significant effects (genotype and treatment) observed in this experiment for SA. Proline is also important for ROS signaling, protects against oxidative stress, and delays aging in plants [39]. In general, the accumulation of stress metabolites (ABA, SA, and proline) in grapevine leaves was related to the duration of drought, as shown by their position in PCA, and was confirmed to be a good marker of water deficit. Only MDA was positioned near the control samples or the samples of the early stress points in the 2-D plot (Figure 3), indicating that the genotypes kept balance with thermal dissipation and did not suffer severe oxidative stress or damage to the photosynthetic apparatus in this experiment [8]. The prompt changes in metabolites observed in this study, particularly ABA, and the delayed accumulation of MDA are consistent with a previous study in which 40% of the significant metabolic changes in grapevines occurred on the fourth day, while activation of ROS metabolism and genes, including those for proline, were up-regulated eight days after the beginning of water deficit [10].

The metabolic response observed here under drought stress suggests an early ABA accumulation and a prominent role of proline, as ABA can induce proline biosynthesis [39]. Although proline accumulation was not genotype driven in this study, WD treatment significantly affected proline accumulation. Proline may induce SA accumulation [40] and was previously shown to accumulate in an SA-dependent manner [41]. On the other hand, lower, or marginal, accumulation of MDA may confirm the antioxidant activity of proline and its negative correlation with MDA content [42].

### 3.2. Selected Gene Expression: ABA Biosynthesis-Related Genes NCED1 and NCED2, and Aquaporin Genes TIP2;1 and PIP2;1

ABA is a metabolite and *NCED1* and *NCED2* genes are related to this metabolite; both can be a good marker at different stages. The genes could be more sensitive and used as markers in early stages of drought stress. ABA and aquaporins are related because ABA can regulate the closure of AQPs by alkalinization of xylem sap and membrane depolarizations [43]. The results showed a predominant down-regulation of the ABA- and AQP-related genes, with the exception of *NCED1* in PMC, and a tendency toward up-regulation in TR and SYL. In this regard, *NCED1* proved to be a better marker than ABA concentration [12], as it could help to identify when the ABA peak occurred under stress conditions, or approximately up to the time when the primary steps of ABA biosynthesis were most pronounced. Our results are in agreement with data published by [44] which reported an increased expression of *NCED1* in leaves of ‘Grenache’ and ‘Shiraz’ grapevines under water deficit. The complete down-regulation of *NCED2*, a key enzyme for de novo biosynthesis of ABA, may indicate that its activity is higher in roots, since its high expression was observed in the self-rooted rootstock 1103P [30]. Saud et al. [45] also found that the root system plays a vital role under drought in bluegrass with pronounced modifications in the anatomy of root tissue. The observed differences in gene regulation depend on genotype sensitivity, duration, and severity of water deficit stress [46,47].

AQP genes, particularly *PIP2*;*1*, have previously been observed to be more up-regulated at early stages of water deficit [15] or after re-watering [15,48]. The down-regulation of AQP genes, as previously observed for TIPs and PIPs [15] in leaves, may indicate a higher rate of closure of aquaporins during moderate to severe water deficit under the influence of high levels of ABA [9]. AQP genes play an important role in root hydraulics under well-watered conditions and a minor role under drought [49]. Results from GR, TR, and SYL indicate a delayed increase in expression of AQPs in response to drought (generally greater after nine than after six days of water deficit treatment). 

## 4. Materials and Methods

### 4.1. Plant Material and Experimentation

Water deficit studies were conducted during 2021 in an experimental greenhouse of the Institute of Adriatic Crops and Karst Reclamation in Split, Croatia (IAC) on four cultivars of *Vitis vinifera* subsp. *vinifera* L.: ‘Plavac mali crni’ (PMC), ‘Istrian Malvasia’ (IM), ‘Graševina’ (GR), and ‘Tribidrag’ (TR) and a *Vitis vinifera* subsp. *sylvestris* genotype IJK88 (SYL). Grapevines were propagated from hardwood *virus free* (*Vitis vinifera* L.) cuttings. Cuttings of TR were from the grapevine collection of the IAC in Split, PMC and GR from the University of Zagreb, Faculty of Agriculture, while cuttings of IM were obtained from Vivai Cooperativi Rauscedo (VCR) Italy. Cuttings of the wild SYL genotype were taken from the natural habitat in Paklenica National Park in Croatia, previously genotyped and confirmed as belonging to *Vitis vinifera* subsp. *sylvestris* [50]. Own-rooted grapevines were grown in 5-L pots in growing medium (soil:humus:perlite = 1:1:1 and the substrate was additionally mixed with quartz sand, 1:1). All plants were watered twice a week to field capacity until the start of the experiment. Nutrient 1/4 strength Hoagland solution was applied once a week for three weeks prior to the experiment, starting when the plants developed the first mature leaf. Temperature, relative humidity, soil water content and vapor pressure deficit were recorded hourly during the drought experiment using an iMETOS Eco-D3 and an online meteorological data system, Field Climate (Pessl Instruments, Weiz, Austria). The average daily temperature during the experimental period was 27 °C. The highest vapor pressure deficit (VPD) of 3.4 kPa was measured on the ninth day of the water deficit treatment (June 9). Average daily relative humidity (RH) was 47%. 

Disease control (against powdery mildew) was regularly applied before water deficit treatment.

Each cultivar consisted of 18 plants arranged in a completely randomized design. The water deficit experiment was conducted from 31 May to 9 June 2021. Nine plants were subjected to the water deficit treatment (WD) via withholding water, while the corresponding controls of nine plants were irrigated to field capacity (WW). At the time of sampling, shoots were ~100 cm long, had 15–20 leaves, and were free of fruit. 

Leaf samples for biochemical and gene expression analyses were collected from individual plants on two different days: 6 June 2021 (day six) and 9 June 2021 (day nine). Samples were collected from the fifth fully developed leaf (day six) and the sixth fully developed leaf (day nine) from the shoot tip of the stressed plants and corresponding controls. Each leaf was divided into two halves with scissors and the petiole and central vein were removed. One half of the leaf was used for metabolite analysis, while the other half was used for gene expression analysis. For each biological replicate, the leaf halves of three plants were divided, providing a total of three biological replicates per treatment and corresponding controls. All collected leaves were snap-frozen in liquid nitrogen (N_2_) and stored at −80 °C. 

### 4.2. Biochemical Analyses

#### 4.2.1. Proline Determination 

Proline concentrations were assayed according to [51], with some modifications. In brief, the samples were lyophilized first, and then extraction was performed using 30 mg of the freeze-dried tissue in 70% *v*/*v* ethanol. Lyophilized tissue (30 mg) was homogenized using a Mixer Mill Retsch MM 400 for 3.5 min at 30 Hz, extracted in 70% *v*/*v* ethanol (1.3 mL), incubated for 30 min at 4 °C, and centrifuged at 16,000× *g* at 4 °C for 10 min. A volume of 100 µL of the extract was mixed with 1000 µL of the reaction mixture (1% *w*/*v* ninhydrin, 60% *v*/*v* acetic acid, and 20% *v*/*v* ethanol), and then heated to 95 °C for 20 min. Proline levels were measured at 520 nm using a UV–VIS spectrophotometer (BioSpec-1601 E, Shimadzu, Kyoto, Japan) and calculated using a standard curve (y = 0.0015x, R^2^ = 0.9991; serial concentrations of proline standard (Sigma–Aldrich, Saint Louis, MO, USA): 0.04, 0.1, 0.2, 0.4, 1.0, 1.5 mM). The proline content was expressed in µmol g^−1^ dw (dry weight). Measurements were performed in three biological replicates. For each biological replicate, three technical replicates were performed.

#### 4.2.2. Lipid Peroxidation (MDA) Measurement

Lipid peroxidation was determined via the malonaldehyde (MDA) method according to [52], with a slight modification. Approximately 30 mg of lyophilized plant leaf tissue was homogenized by a Mixer Mill Retsch MM 400 for 3.5 min at 30 Hz. To the homogenized powder, 1 mL of 0.1% *v*/*v* TCA was added, and the suspension was centrifuged at 13,000 rpm at 4 °C for 10 min. The reaction mixture (400 μL of the obtained supernatant (or 400 μL of 0.1% *v*/*v* trichloroacetic acid, TCA for the blank) and MDA reagent (0.5% *w*/*v* thiobarbituric acid (TBA) in 20% *v*/*v* TCA, Sigma Aldrich, Saint Louis, MO, USA) was incubated at 95 °C for 25 min and centrifuged at 13,000 rpm and 4 °C for 10 min. The absorbance of supernatants was measured at 532 and 600 nm, and the content of MDA was calculated using an extinction coefficient of 155 mM^−1^ cm^−1^. MDA content was expressed as nmol MDA g^−1^ dw. Measurements were performed in three biological replicates. For each biological replicate, three technical replicates were performed.

#### 4.2.3. Salicylic Acid (SA) and Abscisic Acid (ABA)

SA and ABA were purchased from Fluka, and (+)-cis, trans abscisic acid was purchased from Duchefa-Biochemie. The internal isotope-labeled standard salicylic acid-d_6_ was purchased from Sigma-Aldrich, Darmstadt, Germany; (+)-cis, trans abscisic acid-d_6_ from Trc. MiliQ^®^ water (18.2 MW cm^−1^; purified by MiliQ water purification system; Millipore, Bedford, MA, USA); and HPLC gradient-grade methanol (J.T. Baker, Center Valley, PA, USA) were used with analytical-grade formic acid (FA) (Acros Organics, Geel, Belgium) for mobile phase preparation.

Concentrations of SA and ABA in leaves were determined using LC–MS/MS analysis, as previously described [53]. In brief, 30 mg of the powdered freeze-dried leaves were extracted in 1 mL of 10% *v*/*v* methanol containing 1% *v*/*v* acetic acid. A total of 40 μL of salicylic acid-d_6_ and abscisic acid-d_6_ as internal standards (1 μg/mL) was added to each sample, followed by homogenization for 2 min at 30 Hz, and then 60 min equilibration in the cold chamber. After centrifugation (10 min/13,000 rpm), the supernatant was collected and filtered (Acrodisc^®^, LC 13, PVDF, 0.45 nm) into vials. A total of 100 μL of the clear solution was injected into a Zorbax XDP C18 column (75 × 4.6 mm, 3.5 μm particle size) (Agilent Technologies Inc., Palo Alto, CA, USA).

LC–MS/MS analysis was carried out using an Agilent Technologies 1200 series HPLC system equipped with a binary pump, a vacuum membrane degasser, an automated autosampler, and an injector interfaced with a 6420 triple quadropole mass spectrometer with an electrospray ionization source (ESI) (Agilent Technologies Inc., Palo Alto, CA, USA). Solvents for the analysis were 0.1% *v*/*v* formic acid (FA) in water (solvent A) and methanol (solvent B). The gradient was applied as follows: 0 min 50% A, 5–15 min 50% A-0% A, 15–17 min 0% A, 17.1–22 min 60% A. Flow rate was 0.3 mL/min.

The electrospray ionization source was operated in the negative mode, and samples were detected in the multiple reaction monitoring (MRM) mode with a dwell time of 10 ms per MRM transition. The desolvation gas temperature was 350 °C with a flow rate of 6.0 L/min. The capillary voltage was 3.5 kV. The collision gas was nitrogen. The MRM transitions of precursor to product ion pairs were *m*/*z* 263-153 for ABA (quantifying ion), *m*/*z* 263-219 for ABA (qualifying ion), *m*/*z* 137-93 for SA, *m*/*z* 269-159 for ABA-d_6_, and *m*/*z* 141-97 for SA-d_6_. Fragmentor voltages were 100 V for ABA and ABA-d_6_ and 70 V for SA and SA-d_6_. Collision energy was set at 15 V for SA, 12 V for SA-d_6_, 3 V for ABA quantifying and ABA-d_6_, and 2 V for ABA qualifying transition. All data acquisition and processing were performed using Agilent MassHunter software (Agilent Technologies, Inc. 2006–2007, Santa Clara, CA, USA). ABA and SA were calculated based on previously prepared calibration curves as previously described [53] and were presented as ng mg^−1^ dw.

### 4.3. qPCR Analyses of Selected Genes

For total RNA isolation, leaf tissue was homogenized in liquid nitrogen using a mortar and pestle. Then, 50 mg of the homogenized material was pulverized with zirconium oxide beads (Next Advance, Inc., Averill Park, NY, USA) in a Retsch MM400 mixing mill (Retsch GmbH, Haan, Germany) in three cycles of 1 min at 30 Hz. To the homogenized samples obtained, 800 µL of CTAB buffer was added and total RNA was isolated using a Direct-zol^TM^ RNA MiniPrep Kit (Zymo Research, Irvine, CA, USA) according to [54], with slight modifications. DNA was removed using recombinant RNase-free DNase I (Roche Diagnostics GmbH, Mannheim, Germany). Up to 30 μL of total RNA (1 µg) was incubated with 1 U of DNase for 30 min at 37 °C in 1× incubation buffer, followed by the addition of 100 µL of RNA binding buffer from an RNA Clean and Concentrator Kit (Zymo Research, Irvine, CA, USA), and subsequent purification was performed according to the manufacturer’s instructions. The final concentrated DNase-treated RNA was eluted in 10 µL of RNase-free water. RNA integrity was checked before and after DNase I treatment via non-denaturing agarose gel electrophoresis and measuring concentration with a NanoDrop ND-2000 spectrophotometer (NanoDrop Technologies, Wilmington, DE, USA). cDNA was synthesized using the Maxima First Strand cDNA Synthesis Kit for qPCR (Thermo Scientific) according to the manufacturer’s protocol. Reverse transcription was performed with 0.2 μg DNase-treated total RNA in a thermal cycler (Applied Biosystems, Waltham, MA, USA) under the following conditions: 10 min at 25 °C, followed by 15 min at 50 °C, and 5 min at 85 °C.

Gene-specific primers were designed using primer-BLAST [55] or previously published primers were used [15,56,57]. Reference genes were selected according to [58] and validated for normalization using the geNorm [59] and NormFinder tools in Genex software v6.1. All primers used are listed in Table S1. Quantitative PCR was performed in a 96-well optical plate using a 7300 Real-Time PCR System (Applied Biosystems) and universal cycling conditions (10 min 95 °C, 40 cycles of 15 s at 95 °C and 60 s at 60 °C), followed by generation of a dissociation curve to check the specificity of amplification. Reactions contained a Power SYBR Green Master Mix (Applied Biosystems), 300 nM of a gene-specific forward and reverse primer, and 3 μL of 10× diluted cDNA in each 15 μL reaction. The no-template controls (NTC) instead contained 3 μL RNase-free water. For qPCR, three biological replicates were performed in triplicate (technical replicates). PCR amplification efficiencies were calculated using LinRegPCR software v2020.2 [60]. Table S1 shows the amplification efficiencies for the primer pairs we examined. 

### 4.4. Statistical Analysis

Two-way analysis of variance (ANOVA) was used to analyze the effects of genotype, water treatment (WW and WD), and their interactions on the accumulation of stress metabolites in grapevine leaves. A post-hoc Tukey-HSD test was used to separate the means. The significance of differences in biochemical and transcriptomic analyses between the genotype under treatment and the corresponding control was determined using the *t*-test with mean standard error bars (±SE). These data analyses were performed using the STATISTICA v. 14.0 programme (TIBCO Software Inc., Palo Alto, CA, USA)*. p* values < 0.05 were considered statistically significant throughout. Principal component analysis (PCA) was used to group genotypes based on biochemical and gene expression data using mean values in XLSTAT (Addinsoft, 2021, New York, NY, USA). Relative gene expression (fold change) was calculated using the comparative 2^−ΔΔCt^ method [61] with *TRU5* and *TIP4*-*1* as reference genes. Statistical analysis of the data obtained was performed using the *t*-test to determine the differences between the control and treated groups. Results are presented as mean ± SE of four independent experiments.

## 5. Conclusions

The data present evidence on the dynamic response of five *Vitis vinifera* L. genotypes exposed to water withholding for nine days. Prompt metabolite accumulation was observed for ABA, SA and proline, of which ABA had the strongest and earliest signals. Parallel with up-regulation of *NCED1* gene, ABA proved to be a reliable marker for early detection of water deficit in grapevines. Among metabolites, only lipid peroxidation (MDA) had lower values and a delayed response under water deficit which points out significance in prompt adjustment of water and stomatal conductance, cellular osmosis, and antioxidant activity during first days of water deficit in grapevines. Metabolite changes in this study were driven by water regime with a significant impact of genotype. However, additional and combined physiological studies are necessary to obtain a broader picture on genotype adaptation to water deficit.

## Figures and Tables

**Table 1:** Stress responsive metabolites (ABA, SA, proline and MDA) in leaves of four *Vitis vinifera* L. cultivars (cvs.: Plavac mali crni—PMC; Istrian Malvasia—IM; Graševina—GR; Tribidrag—TR) and one wild *V. sylvestris*, SYL) under well-watered (WW) and water deficit (WD) conditions measured at two time points; six and nine days of water deficit treatment.

Day Six											Day Nine									
Metabolites	Genotype						Treatment				Genotype						Treatment			
	PMC	IM	GR	TR	SYL		WW	WD		G × T	PMC	IM	GR	TR	SYL		WW	WD		G × T
ABA	0.42 ^a^	0.75 ^b^	0.46 ^a^	0.39 ^a^	0.41 ^a^	***	0.25	0.73	***	**	0.61	0.71	0.71	0.55	0.76	ns	0.27	1.05	***	ns
SA	18.06 ^a^	19.64 ^a^	43.96 ^b^	30.03 ^ab^	14.60 ^a^	***	21.00	27.85	*	ns	16.04 ^a^	35.50 ^ab^	48.94 ^b^	23.42 ^ab^	16.10 ^a^	**	19.92	34.69	**	ns
Proline	4.10	5.64	4.11	2.66	4.39	ns	3.03	5.43	**	*	11.48	10.29	8.58	7.57	9.91	ns	3.25	16.36	***	ns
MDA	553.63 ^c^	355.14 ^a^	414.68 ^ab^	558.29 ^bc^	455.73 ^abc^	***	457.40	468.66	ns	ns	499.67 ^b^	382.70 ^ab^	471.20 ^ab^	317.54 ^a^	279.54 ^a^	*	503.78	297.40	***	ns

Data were analyzed using a two-way ANOVA with genotype (G) and treatment (T) as main factors, and their interaction (G × T). When a factor was tested as significant (* *p* < 0.05; ** *p* < 0.01; *** *p* < 0.001; otherwise ns, non-significant), Tukey post-hoc test was used to separate the means; different letters indicate significant differences between the genotypes.

## Data Availability

The data presented in this study are available in [Biochemical response and gene expression to water deficit of four Croatian grapevine cultivars (*Vitis vinifera* L.) and one *Vitis sylvestris* article and corresponding Supplementary Materials].

## Supplementary Materials

The supporting information can be downloaded at: https://www.mdpi.com/article/10.3390/plants12193420/s1, Table S1: Primers in qPCR experiments, Figure S1: The contents of (A) abscisic acid (ABA), (B) salicylic acid (SA), (C) proline and (D) malondialdehyde (MDA) measured in leaves of five grapevine genotypes: cvs.: ‘Graševina’ (GR), ‘Istrian Malvasia’ (IM), ‘Plavac mali crni’ (PMC), ‘Tribidrag’ (TR) and wild accession of *Vitis sylvestris* (SYL). Measurements were performed at two time points (day six and day nine) from the beginning of water deficit (WD) treatment. Graph columns and control values represent corresponding mean values summarizing both time points. Genotype is represented with three replicates (n = 3) unless indicated otherwise. Controls are normalized to the value 1.
