# Supplementary material for: Biochemical Response and Gene Expression to Water Deficit of Croatian Grapevine Cultivars (Vitis vinifera L.) and a Specimen of Vitis sylvestris
Source: Plants (Basel). 2023 Sep 28;12(19):3420. doi: 10.3390/plants12193420 (PMC10575188; doi:10.3390/plants12193420)
Supplement: Supplementary file 1 [file plants-12-03420-s001.zip › plants-2585257-supplementary.pdf]

Biochemical Response and Gene Expression to Water Deficit of Croatian Grapevine Cultivars (*Vitis vinifera* L.) and a Specimen of *Vitis sylvestris*

Katarina Lukšić [1,†], Ana Mucalo [1,†], Ana Smolko [2], Lidija Brkljačić [2], Luka Marinov [1], Katarina Hančević [1], Maja Ozretić Zoković [1], Marijan Bubola [3], Edi Maletić [4,5], Jasminka Karoglan Kontić [4,5], Marko Karoglan [4], Branka Salopek-Sondi [2] and Goran Zdunić [1,*]

[1] Institute for Adriatic Crops and Karst Reclamation, Put Duilova 11, 21000 Split, Croatia; katarina.luksic@krs.hr (K.L.); ana.mucalo@krs.hr (A.M.); luka.marinov@krs.hr (L.M.); katarina.hancevic@krs.hr (K.H.); maja.ozretic@krs.hr (M.O.Z.)

[2] Ruđer Bošković Institute, Bijenička 54, 10000 Zagreb, Croatia; ana.smolko@irb.hr (A.S.); lidija.brkljacic@irb.hr (L.B.); branka.salopek.sondi@irb.hr (B.S.-S.)

[3] Institute of Agriculture and Tourism, Karla Huguesa 8, 52440 Poreč, Croatia; marijan@iptpo.hr

[4] Department of Viticulture and Enology, Faculty of Agriculture, University of Zagreb, Svetošimunska Cesta 25, 10000 Zagreb, Croatia; emaletic@agr.hr (E.M.); jkkontic@agr.hr (J.K.K.); mkaroglan@agr.hr (M.K.)

[5] Centre of Excellence for Biodiversity and Molecular Plant Breeding, Svetošimunska Cesta 25, 10000 Zagreb, Croatia

* Correspondence: goran.zdunic@krs.hr; Tel.: +385-21-434-453.

† These authors contributed equally to this work.

Table S1. Primers in qPCR experiments

Primer name	Sequence 5′ → 3′	Efficiency of amplification	Gene ID (database)
*VvSAND*_fw [58]	CAACATCCTTTACCCATTGACAGA	1.938	GSVIVT01025191001 (*Vitis* Genoscope)
*VvSAND*_rev [58]	GCATTTGATCCACTTGCAGATAAG		
*VvN227*_fw fw [58]	GAAGATGAGGAGGCGGAAAGAC	1.959	GSVIVT01026115001 (*Vitis* Genoscope)
*VvN227*_rev fw [58]	TAGTTGAGATATGCGCTGATGATGC		
*VvTIP4-1*_fw fw [58]	CTCGCAAGCGTTCCATTCTCAA	1.912	GSVIVT01037896001 (*Vitis* Genoscope)
*VvTIP4-1*_rev fw [58]	AAAAACCATCTCCGGCAAGTGTG		
*VvTRU5*_fw fw [58]	CAATGTACGAGCTTTATGACCCATC	1.938	GSVIVT00020074001 (*Vitis* Genoscope)
*VvTRU5*_rev fw [58]	CAGTTGATCTTGTTGTTGTTTCCAG		
*VvNCED1*_fw [56,57]	GAGACCCCAACTCTGGCAGG	1.917	VIT_19s0093g00550 (PN40024 12X V1)
*VvNCED1*_rev [56,57]	AAGGTGCCGTGGAATCCATAG		
*VvNCED2*_fw fw [56]	AGTTCCATACGGGTTTCATGGG	1.94	VIT_10s0003g03750 (PN40024 12X V1)
*VvNCED2*_rev fw [56]	CCATTTTCCAAATCCAGGGTGT		
*VvTIP2;1*_fw	AGCCCTTGCTTTGGTTCAAGC	1.881	EF364439 (NCBI)
*VvTIP2;1*_rev	GTGGCTGCACATCACCAACC		
*VvPIP2;1*_fw [15]	CAGGAGCACCACTCATGTATG	1.975	VIT_13s0019g04280, AY823263 (PN40024 12X V1, NCBI)
*VvPIP2;1*_rev [15]	TCATGCCCTCATACATATCAATAAC		

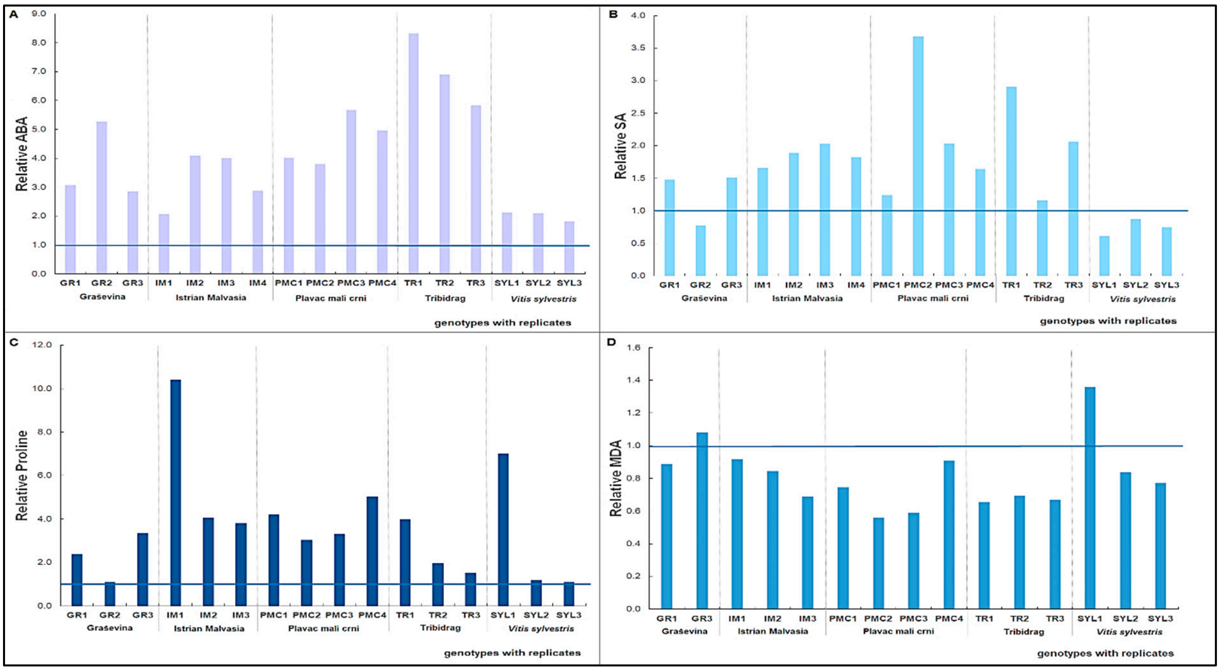

Figure S1. The contents of (A) abscisic acid (ABA), (B) salicylic acid (SA), (C) proline and (D) malondialdehyde (MDA) measured in leaves of five grapevine genotypes: cvs.: 'Graševina' (GR), 'Istrian Malvasia' (IM), 'Plavac mali crni' (PMC), 'Tribidrag' (TR) and wild accesion of *Vitis sylvestris* (SYL). Measurements were performed at two time points (day six and day nine) from the beginning of water deficit (WD) treatment. Graph columns represent corresponding mean values summarizing both time points. Genotype is represented with three replicates (n = 3) unless indicated otherwise. Controls are normalized to the value 1.
